# Effect of daridorexant on sleep architecture in patients with chronic insomnia disorder: a pooled post hoc analysis of two randomized phase 3 clinical studies

**DOI:** 10.1093/sleep/zsae098

**Published:** 2024-04-22

**Authors:** Tobias Di Marco, Ina Djonlagic, Yves Dauvilliers, Kolia Sadeghi, David Little, Alexandre N Datta, Jeffrey Hubbard, Göran Hajak, Andrew Krystal, Antonio Olivieri, Liborio Parrino, Corey B Puryear, Gary Zammit, Jacob Donoghue, Thomas E Scammell

**Affiliations:** Idorsia Pharmaceuticals Ltd, Allschwil, Switzerland; Department of Clinical Research, University of Basel, Schanzenstrasse, Basel; Department of Neurology, Beth Israel Deaconess Medical Center, Boston, MA, USA; Centre National de Référence Narcolepsie, Unité du Sommeil, CHU Montpellier, Hôpital Gui-de-Chauliac, Université de Montpellier, INSERM INM, Montpellier, France; Beacon Biosignals, Inc., Boston, MA, USA; Beacon Biosignals, Inc., Boston, MA, USA; Zentrum für Schlaf- und Chronomedizin der Basler Universitätskliniken, University Children’s Hospital Basel, Basel, Switzerland; Idorsia Pharmaceuticals Ltd, Allschwil, Switzerland; Social Foundation Bamberg, Department of Psychiatry, Psychosomatic Medicine and Psychotherapy, Bamberg, Germany; Departments of Psychiatry and Neurology, University of California, San Francisco, CA, USA; Idorsia Pharmaceuticals Ltd, Allschwil, Switzerland; Department of Medicine and Surgery, University of Parma, Parma, Italy; Beacon Biosignals, Inc., Boston, MA, USA; Clinilabs Drug Development Corporation, New York, NY, USA; Beacon Biosignals, Inc., Boston, MA, USA; Department of Neurology, Beth Israel Deaconess Medical Center, Boston, MA, USA

**Keywords:** insomnia, EEG analysis, neurobiology of sleep and arousal, sleep–wake mechanisms, sleep–wake physiology, sleep and the brain, sleep spindles

## Abstract

**Study Objectives:**

Post hoc analysis to evaluate the effect of daridorexant on sleep architecture in people with insomnia, focusing on features associated with hyperarousal.

**Methods:**

We studied sleep architecture in adults with chronic insomnia disorder from two randomized phase 3 clinical studies (Clinicaltrials.gov: NCT03545191 and NCT03575104) investigating 3 months of daridorexant treatment (placebo, daridorexant 25 mg, daridorexant 50 mg). We analyzed sleep–wake transition probabilities, EEG spectra, and sleep spindle properties including density, dispersion, and slow oscillation phase coupling. The wake EEG similarity index (WESI) was determined using a machine learning algorithm analyzing the spectral profile of the EEG.

**Results:**

At month 3, daridorexant 50 mg decreased wake-to-wake transition probabilities (*p* < .05) and increased the probability of transitions from wake-to-N1 (*p* < .05), N2 (*p* < .05), and REM sleep (*p* < .05), as well as from N1-to-N2 (*p* < .05) compared to baseline and placebo. Daridorexant 50 mg decreased relative beta power during wake (*p* = .011) and N1 (*p* < .001) compared to baseline and placebo. During the wake, relative alpha power decreased (*p* < .001) and relative delta power increased (*p* < .001) compared to placebo. Daridorexant did not alter EEG spectra bands in N2, N3, and REM stages or in sleep spindle activity. Daridorexant decreased the WESI score during wake compared to baseline (*p* = .004). Effects with 50 mg were consistent between months 1 and 3 and less pronounced with 25 mg.

**Conclusions:**

Daridorexant reduced EEG features associated with hyperarousal as indicated by reduced wake-to-wake transition probabilities and enhanced spectral features associated with drowsiness and sleep during wake and N1.

**Clinical Trials:**

ClinicalTrials.gov NCT03545191: study to assess the efficacy and safety of ACT-541468 (daridorexant) in adult and elderly participants with insomnia disorder. URL: Study Details | study to assess the efficacy and safety of ACT-541468 (daridorexant) in adult and elderly participants with insomnia disorder | ClinicalTrials.gov ClinicalTrials.gov NCT03575104:

study to assess the efficacy and safety of ACT-541468 (daridorexant) in adult and elderly participants who experience difficulties sleeping. URL: study details | study to assess the efficacy and safety of ACT-541468 (daridorexant) in adult and elderly participants who experience difficulties sleeping | ClinicalTrials.gov

## Graphical Abstract

**Graphical Abstract:**
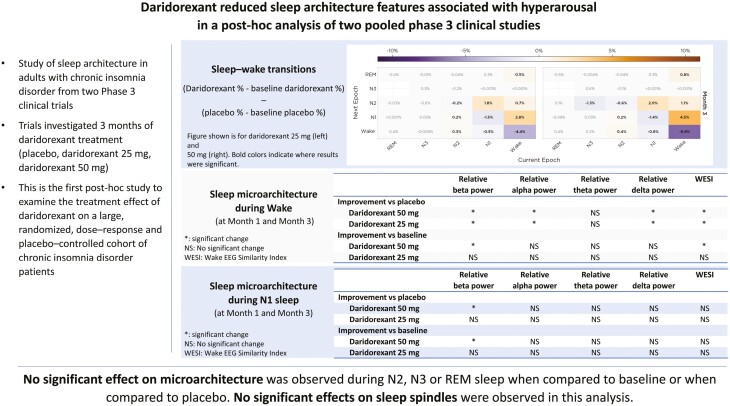

**Statement of Significance**

This is the first study to examine the treatment effect of a dual orexin receptor antagonist (DORA), daridorexant, on a large, randomized, dose–response, and placebo-controlled cohort of 1466 chronic insomnia disorder patients. The results from this post hoc analysis provide evidence that daridorexant reduces features associated with hyperarousal, a well-characterized aspect of this disorder. We observed significant increases in probabilities to transition from wake to sleep, in addition to significant modifications of EEG spectral power in beta, alpha, and delta bands particularly at the higher 50 mg dose, both after 1 and 3 months of treatment. Taken together, these treatment-related changes underlie a possible mechanism for how DORAs, such as daridorexant, improve insomnia symptoms by reducing hyperarousal.

Chronic insomnia disorder is the most prevalent sleep disorder, affecting approximately 10% of the global population [1] and is associated with substantial impairment in quality of life [2] Although almost exclusively diagnosed through self-reported complaints of sleep and daytime functioning [3], objective measures like polysomnography (PSG) can offer valuable insights into the pathophysiology of insomnia [3, 4]. One of the leading hypotheses suggests that chronic insomnia disorder is characterized by a state of hyperarousal that can be defined as a persistent state of cognitive, emotional, physiological, or cortical arousal [5], impacting both daytime and nighttime function [6, 7]. Numerous studies in patients with insomnia have reported increased high-frequency EEG activity during sleep compared to individuals without insomnia [8–10], as well as more time spent in long wake bouts over an 8-hour PSG recording [11]. These findings are corroborated in a companion paper in which we demonstrate that participants with insomnia had an increase in neurophysiological arousal across various EEG features during sleep, when compared to participants without insomnia. These EEG findings are consistent with a positron emission tomography study showing hypermetabolism in the hypothalamus and the relevant efferent projections of arousal networks, as well as excessive cortical activity, during sleep in patients with insomnia compared to controls without insomnia, supporting the presence of hyperarousal in insomnia [12]. Therefore, analyzing the effects of pharmacological and non-pharmacological interventions for chronic insomnia disorder on those sleep architecture features indicative of hyperarousal, can help guide treatment choices and outcomes.

Most guidelines for the treatment of chronic insomnia recommend cognitive behavioral therapy for insomnia (CBT-I) as the first-line approach for disease management [13–15]. CBT-I can reduce hyperarousal in patients with insomnia, as indicated by reductions in high-frequency EEG spectral power (beta power), which is associated with wakefulness [16]. This decrease in beta activity, along with enhancements in sleep quality, has been observed when comparing post-treatment outcomes to baseline measurements taken prior to starting CBT-I [16]. However, challenges associated with using CBT-I, such as limited accessibility, can reduce its use in clinical practice [17, 18].

This leads to the frequent and long-term use of sleep-promoting medications, despite long-term treatment risks such as tolerance and dependence [13, 15, 19]. Hypnotics with pharmacological activity at α1-containing GABAA receptors such as zolpidem reduce sleep onset latency and increase sleep time but effects on EEG spectral power differ across studies [20]. For example, a reduction in alpha activity has been observed in healthy volunteers without sleep disorders, while beta activity increased in the first hour of treatment [21]. Other studies identified that individuals with insomnia showed more delta power during the initial two hours of sleep while treated with zolpidem, but this effect did not persist throughout the night, consistent with the known pharmacokinetic profile of this compound [22]. Taken together, this underlines the inconsistent evidence regarding zolpidem’s impact on EEG markers of hyperarousal [20].

In recent years, dual orexin receptor antagonists (DORAs), which block both types 1 and 2 orexin receptors (OXR1 and OXR2, respectively) have become available for treating chronic insomnia disorder. The orexin/hypocretin system is known to play a key role in stabilizing wakefulness and promoting arousal [23–25]. DORAs dampen these arousing effects of the orexin system and promote sleep. There is limited evidence on the effect of DORAs in modulating sleep architecture and continuity. For example, suvorexant and lemborexant have been shown to increase total sleep time (TST), as well as the time spent in all sleep stages, in patients with insomnia [26, 27]. While suvorexant was found to reduce long wake bouts [28] it only minimally modulated sleep microarchitecture by decreasing gamma and beta EEG activity after 1 night of treatment, but no differences relative to placebo were observed after 1 and 3 months of treatment [26]. Daridorexant, the most recent DORA available for patients with insomnia, was shown to improve sleep onset, sleep maintenance, subjective TST, and daytime functioning in two pivotal 3-month clinical studies [29]. Furthermore, additional studies found that daridorexant reduced both the number and duration of long awakenings across the entire 8-hour night [11]. The increase in sleep duration did not alter the proportion of time spent in NREM and REM sleep stages compared to placebo [29].

The current study investigated whether daridorexant, administered at the two effective doses of 25 and 50 mg for 3 months, can reduce EEG features associated with hyperarousal, in patients with chronic insomnia disorder. We performed a post hoc analysis of two, 3-month, placebo-controlled, double- blind, randomized studies [29] to assess daridorexant’s effects on sleep architecture and continuity including sleep–wake transitions, changes to EEG spectral power content, and changes to sleep spindle features. Data from two independent phase 3 trials with identical study design and conduct were pooled to increase the sample size and provide a more robust analysis. The analysis also incorporated a novel machine learning-derived index of the similarity between a given period of the EEG and the EEG activity during wake, called wake EEG similarity index (WESI).

## Materials and Methods

### Dataset

We analyzed polysomnograms (PSGs) from two phase 3, placebo-controlled randomized studies that evaluated the safety and efficacy of daridorexant (10, 25, and 50 mg) in adult and elderly patients with chronic insomnia disorder (ClinicalTrials.gov identifier NCT03545191 and NCT03575104). Sample size calculations of the original studies were based on a two-sample Z test based on phase 2 dose-finding studies [30, 31], ensuring the study was sufficiently powered and type 1 error rate controlled, which led to the inclusion of at least 900 participants [29]. For the current analysis, the cohorts randomized to placebo and daridorexant 25 mg in both studies were pooled and compared to the cohort randomized to 50 mg in study NCT03545191. Patients receiving placebo and those with 25 mg were pooled between the two studies, as these were identical in design, patient population (participants with insomnia disorder according to DSM-5), inclusion and exclusion criteria, as well as treatment protocols. Furthermore, measured outcomes showed consistency in efficacy and safety between the two phase 3 clinical trials [29]. The group randomized to daridorexant 10 mg was not included in the analysis as this dose did not improve the primary and secondary endpoints on either PSG-derived or subjective sleep measures in the phase 3 study: NCT03575104 when compared to placebo [29].

### Study design

Full details of the study design, as well as the primary study results, have been reported elsewhere [29]. In brief, these two phase 3, double-blind, placebo-controlled, parallel-group clinical trials randomized patients (1:1:1) to receive daridorexant 50 mg, daridorexant 25 mg or placebo or daridorexant 25 mg, daridorexant 10 mg or placebo taken every evening, orally, approximately 30 minutes before going to bed, for 12 weeks. The double-blind treatment period was preceded by a single-blind placebo run-in period (13–24 days) and followed by a 7-day single-blind, placebo run-out period. Randomization was stratified by age (<65 and ≥65 years) and treatment allocation was done using an interactive response technology system. The studies were conducted between May 2018 and May 2020 in 156 sites in 18 countries, in accordance with principles of the Declaration of Helsinki, the International Conference on Harmonization Guideline for Good Clinical Practice, and local regulations. The studies were successfully completed after the required number of participants was recruited and all participants reached the end of the study visit. No major changes were performed to the methods in the original protocol including the data collection and trial outcomes, and there was no planned interim analysis. The protocol was approved by institutional review boards or independent ethics committees, and all patients provided written informed consent. The full study protocol can be obtained in the supplement of the original publication [29].

### Study participants

All study patients randomized in the two studies to daridorexant 25 mg, 50 mg, or placebo were included in the analyses reported here. In brief, all participants were diagnosed with insomnia disorder per the Diagnostic and Statistical Manual of Mental Disorders, fifth edition (DSM-5) criteria [3], and reported moderate to severe insomnia symptoms at baseline based on Insomnia Severity Index scores ≥ 15 [32], and showed difficulty falling asleep (latency to persistent sleep [LPS] ≥ 20 minutes) and maintaining sleep (wake after sleep onset [WASO] ≥ 30 minutes), as well as reduced total sleep time (TST < 420 minutes) on PSG nights. Patients were excluded if they presented with: moderate to severe sleep apnea (apnea–hypopnea index ≥ 15/hour and/or oxygen saturation < 80%), alcohol or drug misuse, or any other acute or unstable psychiatric condition (including but not restricted to anxiety disorder, major depression, bipolar disorder, schizophrenia, and obsessive-compulsive disorder) diagnosed by the Mini International Neuropsychiatric Interview [33] or that required pharmacological treatment.

### Polysomnography

Baseline comprised both PSG nights of the placebo run-in period. During the double-blind treatment phase, patients underwent two consecutive PSG nights at the end of months 1 and 3. Data from all four of these acquisition times were used as separate data points. EEG recordings were high-pass filtered at 0.5 Hz in order to normalize across different acquisition and prefiltering settings that were present at different recording sites. For brevity and simplicity, we report this analysis for central electrodes (C3 and C4) only, as analysis of the other four standard PSG channels (F3, F4, O1, and O2) yielded very similar results. PSGs were scored according to the American Academy of Sleep Medicine Guidelines [34]. A total of 81 patients with insomnia were not included from the original dataset due to failure to extract valid EEG features (e.g. due to interrupted recordings).

### Outcomes

#### Sleep–wake stage transitions.

To analyze the dynamics of sleep stages during the period between lights off and lights on, the likelihood of transitioning between the 5 stages, including wake, was evaluated. Specifically, we counted the number of transitions from one sleep stage in a 30-second epoch to another (possibly the same) sleep stage in the next 30-second epoch, producing a 5-by-5 matrix of counts for each recording. This was modeled as a first-order Markov process as used previously [35].

#### Spectral analysis.

We estimated band powers using multi-taper spectral density estimation [36]. Spectral features were derived for four frequency bands (delta: 0.5–4 Hz, theta: 4–8 Hz, alpha: 8–12 Hz, and beta: 12–30 Hz) [37], computing an average power for each band across all epochs of a given sleep stage. First, we rejected EEG epochs with sweating or movement artifacts. We then calculated the root-mean-squared amplitude for each 3-second segment of the recording, and segments with root-mean-squared error ≤ 1 or ≥ 250 μV were considered artifacts and excluded from downstream analyses. These thresholds were selected to be relatively conservative as they fall well outside the physiologic range (physiological amplitudes fall roughly between 10 and 100 μV [38]). Tapering used 2-second windows with 1-second overlap. By leveraging multiple, orthogonal measurements (multi-taper windows) for each spectral bin, the method aims to reduce bias and variance in spectral-power estimates.

The four band powers in the 2-second windows were averaged to 30-second windows. Given the high variance of absolute power found across recording sites, analyses were focused on the relative power in four bands; for each power band (delta, theta, alpha, and beta), the relative power was computed by dividing the absolute power in each band by the total power of these four bands within the same 30-second window. The 30-second windows were then aggregated to the sleep stage by taking the mean across all epochs of each stage (i.e. N1, N2, N3, REM, and wake) and each channel (C3 and C4). These spectral features were then log-transformed to reduce skew.

#### Spindle analysis.

Spindle features were calculated using the open-source Luna package (version 0.23; https://zzz.bwh.harvard.edu/luna/ref/) following the methods from Purcell et al. [39] To maintain consistency with the use of the Luna spindle analysis package, artifact rejection for EEG signals followed the conventions described in the Luna package. Briefly, we resampled signals to 128 Hz, low-pass filtered them at 35 Hz, and removed artifacts by computing power within the delta band, rejecting epochs that had more than 2.5 the average delta power in a 15-epoch sliding window.

After artifact removal, spindles were detected within all N2 stages by first convolving a Morelet wavelet (13.5 Hz) over the signal and then smoothing the convolution’s magnitude using a sliding window of 0.1 seconds. Spindles were detected from this convolution by thresholding: at least 0.3 seconds had to be over 4.5 times the mean of all N2 epochs, and in a 0.5-second window around this region, power had to be at least twice this N2 epoch mean. These putative spindles were merged if they fell within 0.5 seconds of one another, and any that lasted longer than 3 seconds were rejected. This basic approach to spindle validation, via Morlet wavelets, has been validated against manual spindle annotation [40].

Since sleep spindles are known to couple with slow oscillations during N2 sleep, we detected slow oscillations by: (1) low-pass filtering the entire signal at 4.5 Hz (note that the signal was already high-pass filtered at 0.5 Hz to reduce site-to-site variability) and (2) within the N2 stage, marking all consecutive zero-crossings that fall between 0.8 and 2 seconds in length as a slow oscillation.

Having detected both spindles and slow waves, we computed the following four features (spindle density, spindle dispersion, slow oscillation phase at spindle peak for fast and slow spindles) per channel (C3 and C4) for a total of eight unique features. Note that, because the underlying data are discrete events and can be spatially sparse, not all of these features can be sensibly averaged across channels. Two of the features were computed for all spindles (total range of 11–15 Hz): density (spindle count per minute) and dispersion. We calculated spindle dispersion (how variable spindle counts are across 30-second epochs) by dividing the variance of the spindle counts across epochs of N2 sleep by the average number of spindles across epochs of N2 sleep. Slow oscillation phase at the spindle peak was calculated separately for fast (≥13–15 Hz) and slow (11–<13 Hz) spindles for each channel. For spindles that occurred during a slow oscillation, we compared the peak of the spindle to the start and end of the related slow oscillation, reporting when the peak occurred relative to these two positions as an angle between 0°C and 360°C. The start of the SO was defined as the preceding zero-crossing from positive to negative (relative to the spindle start), and the end was the subsequent such zero-crossing.

#### Wake EEG similarity index.

Informed by the odds-ratio product introduced by Younes et al. [41], we developed the WESI. The objective of this machine learning approach was to place EEG patterns during segments of sleep on a continuum from more to less wake-like. After training, WESI produces a value ranging from 0 (most sleep-like) to 1 (most wake-like) for every 3-second segment of EEG. While the output of WESI is continuous, WESI was trained by labeling all segments that occurred during a sleep stage (i.e. N1, N2, N3, or REM) as 0 and all segments that occurred during a Wake period during the night as 1. The spectral power in the delta, theta, alpha and beta frequency bands was computed for every 3-second window and transformed into model features in the following steps: (1) divided by the sum of powers delta + theta + alpha + beta to obtain the relative power in each band, (2) logit-transformed the relative powers: *log*(*x*/(1 – *x*)), and (3) computed the z-score for each WESI feature as estimated by the training set data.

The set of 3-second segments was randomly split into training and testing sets (80% training data/ 20% test data). The same model as reported in our companion paper (submitted) was used, this included participants from Beacon Clinico-PSG Database (a private database from the Massachusetts General Hospital), the Sleep Heart Health Study, and randomized participant recordings from the first visit of the present study’s data. During our statistical analysis, WESI values were first logit-transformed. We report model coefficients and confidence intervals along a linear scale. To compute linear-scale means and the confidence intervals, we used a finite difference approximation (validated by a bootstrap of 10 000 samples for the CI of the most extreme mean to differ by less than 0.001).

### Statistical analysis

We assessed statistical significance using linear mixed-effects regression models for spectral, spindle, and WESI feature types.

For sleep stage transition counts, many recordings lacked some of the transitions. When necessary, we used a Hurdle model (see below for further discussion of this model design) when at least 1.5% of recordings contained the transition (transitions that occurred in fewer than 1.5% of recordings were not modeled at all) and no more than 98.5% of the recordings contained the transition; otherwise, we used a Generalized Mixed Effects model.

All models used the same covariates: study ID to indicate which phase 3 trial, treatment to indicate placebo (0), 25 mg (25), 50 mg (50), Month to indicate baseline/run-in (0), first month of treatment (1) or third month of treatment (3), age (centered at 50 years old), sex (female coded + 1 and male coded as −1), and night to indicate first night or second night. All models had random intercept effects for participants and sites. A null and main effect model were used, with the following formulas: null model:


$$\begin{array}{l} feature=1+age⁎\;sex⁎month⁎night⁎study\_id+(1|study\_subject\_id)+(1|site) \end{array}$$

main effect model:


$$\begin{array}{l} feature=1+age⁎sex⁎month⁎night⁎study\_id+([month⁎night\;]\;⁎treatment)+(1|study\_subject\_id)+(1|site) \end{array}$$

The statistical significance of effects of treatment vs. placebo and effect of month versus baseline was determined using the main effect model. For treatment to have a significant effect on a given feature, the likelihood ratio test between the main effect and the null models for that feature had to be significant after false discovery rate correction for multiple comparisons across features within each feature type (spectral, spindle, WESI, and sleep stage transition counts), and in addition, the model coefficient (or linear combinations thereof) representing the difference in question (e.g. 50 mg—placebo at month 3) had to be significantly different from zero with a *p*-value < .05.

Sleep stage transitions were modeled with main effect and null models. In the case of sleep-stage transitions observed in more than 1.5% and fewer than 98.5% of recordings, we employed a hurdle model. In each hurdle model, there were two stages: an initial model predicting the probability that a given transition is observed zero times in a recording (the zero-count model), and a second model, conditioned on a non-zero value, predicting the non-zero values (the non-zero-count model). For transitions occurring in fewer than about 98.5% of recordings, it was found that regular generalized mixed-effects models could not adequately fit the number of zero and non-zero counts simultaneously, whereas the Hurdle models could. Both stages of Hurdle models employed a generalized mixed-effect linear regression model with a logistic link function: the zero-count modeled a single observation (zero vs. non-zero) and the second modeled all counts for the non-zero observations. The regression models in both stages of the hurdle model employed the same covariate structure as used above for the other features.

## Results

### Patient baseline characteristics prior to treatment

We analyzed PSG data from patients randomized to either placebo, 25 mg, or 50 mg daridorexant, for 3 months. Prior to treatment (or placebo), groups were balanced for age (see Mignot et al. [29] details), with 67% of patients being female, having a mean WASO ranging from 95 to 106 minutes, a mean LPS ranging from 64 to 70 minutes, and an objective TST between 311 and 324 minutes. Finally, there were no major differences across groups in the proportion of and duration of time spent in each sleep stage for N1, N2, N3, and REM sleep (Table 1). All safety events were reported in the primary publication by Mignot et al. Overall incidence of adverse events was comparable between treatment groups.

**Table 1.** Average Values (SD Standard Deviation) Across the Two PSGs at Baseline

	Treatment group			
	Total^*^	Placebo	25 mg	50 mg
Number of participants	1466	586	584	296
Age, years (SD)	55 (15)	56 (15)	56 (15)	55 (15)
Sex %, female	67	65	66	68
Sleep efficiency (SD)	66 (15)	65 (15)	66 (15)	68 (13)
TST, minutes (SD)	316 (74)	311 (76)	317 (74)	324 (73)
LPS, minutes (SD)	68 (50)	70 (53)	68 (50)	64 (45)
WASO, minutes (SD)	102 (52)	106 (55)	102 (52)	95 (47)
N1 sleep, minutes (SD)	36 (2)	36 (31)	36 (20)	35 (20)
N2 sleep, minutes (SD)	178 (53)	177 (54)	178 (53)	182 (52)
N3 sleep, minutes (SD)	40 (32)	38 (31)	41 (32)	43 (34)
REM sleep, minutes (SD)	62 (26)	61 (26)	62 (26)	64 (25)
Awake, minutes (SD)	162 (72)	168 (74)	162 (72)	151 (65)
% N1 sleep (SD)	7.5 (4.1)	7.5 (4.3)	7.4 (4.0)	7.5 (4.1)
% N2 sleep (SD)	37.3 (10.8)	36.9 (11.1)	37.1 (10.8)	38.3 (10.1)
% N3 sleep (SD)	8.4 (6.6)	7.9 (6.4)	8.6 (6.6)	9.0 (7.0)
% REM sleep (SD)	13.0 (5.4)	12.6 (5.4)	13.0 (5.5)	13.5 (5.1)
% Awake (SD)	33.9 (14.8)	35.0 (15.3)	33.8 (14.9)	31.7 (13.3)

For PSG-derived values, baseline is the mean of two consecutive placebo run-in PSG nights. Results are shown for each treatment group whereas 25 and placebo have been pooled across studies. The “total” column shows the patients’ characteristics for all three treatment groups combined.

^*^81 patients were excluded from the analysis due to the inability to extract valid EEG information.

### Effects of daridorexant on sleep–wake transition probabilities

**Figure 1.**
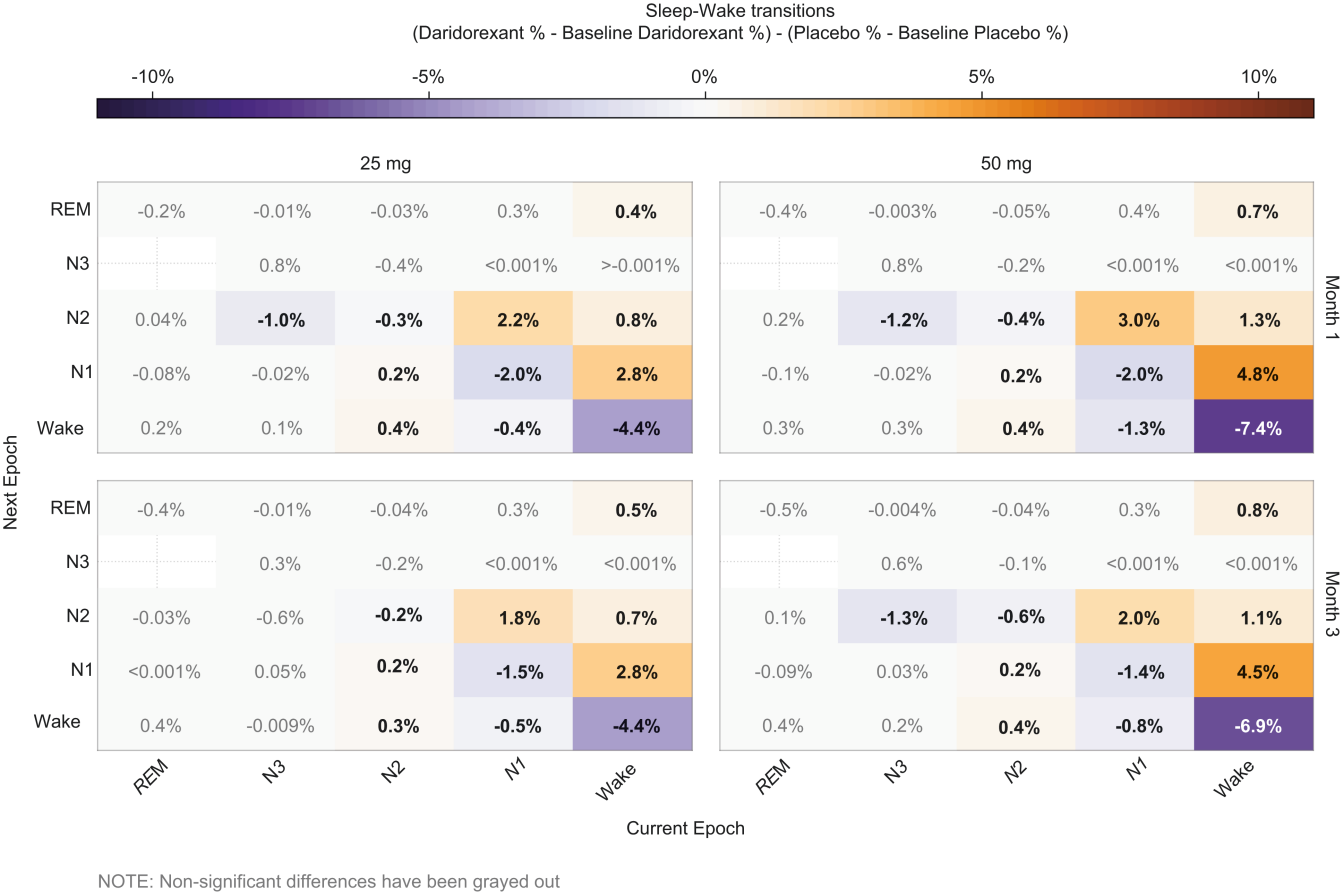
Sleep–Wake transitions: each box shows the change-from-baseline value at months 1 and 3 (between 0 and 1). Statistical significance (*p* < .05) is denoted by the text color, where the gray text indicates no statistical significance. Cool colors denote a decrease, and warm colors denote an increase. Blank box indicates an absence of that sleep–wake transition in either group.

Placebo-controlled changes from baseline to month 3 in transition probability from wake-to-wake decreased by 6.9% and 4.4% with daridorexant 50 mg and 25 mg doses respectively (*p* < .05; Figure 1). Placebo-controlled changes from baseline to month 3 in the transition probability from wake-to-N1, wake-to-N2, and wake-to-REM, increased (*p* < .05) by 4.5% and 2.8%, 1.1% and 0.7%, and 0.8% and 0.5% for daridorexant 50 and 25 mg, respectively. In addition, placebo-controlled probabilities to transition from N1-to-wake and N1-to-N1, decreased (*p* < .05) by 0.8% and 0.5%, and 1.4% and 1.5% with daridorexant 50 and 25 mg doses, respectively, while probabilities to transition from N1-to-N2 increased (*p* < .05) by 2.0% and 1.8% with daridorexant 50 and 25 mg doses at month 3. Furthermore, no significant changes in the probability of transitioning to N3 from any sleep/wake stage in either the 25 or 50 mg dose groups were observed. Results at month 1 were similar to those observed at month 3 (Figure 1). Of note, absolute numbers in sleep/wake stage probability transitions followed similar trends (Supplementary Material S1).

### Effects of daridorexant treatment on EEG spectral and spindle features

**Figure 2.**
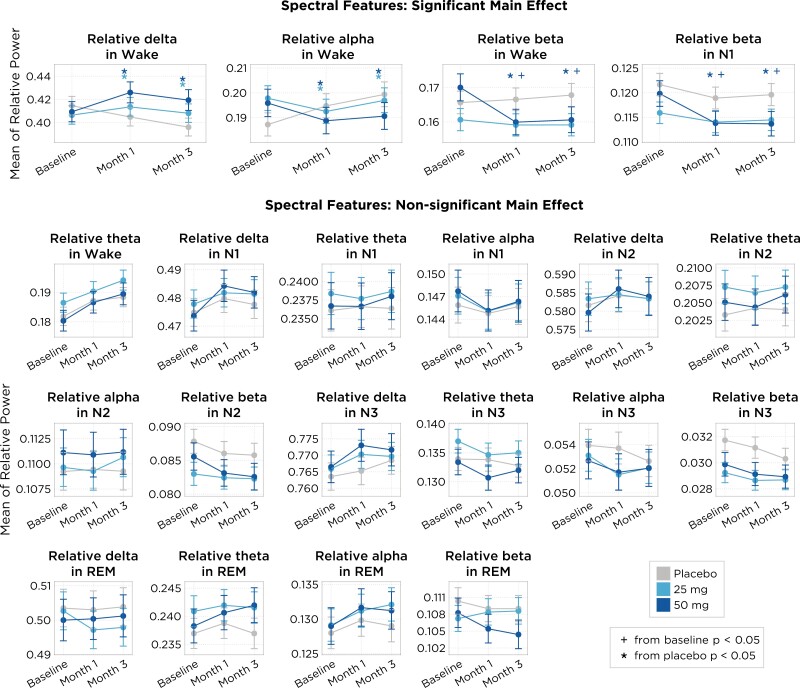
Shown are the mean (points) and 95% CI (shaded region) at each time point (*x*-axis) and treatment arm (colors). The main effect model shows either a statistically significant effect (*p* < .05), indicating that the treatment impacts the feature of interest, or a lack of statistical significance, suggesting that the treatment does not have a measurable impact; both outcomes are adjusted for covariates such as age, sex, month, and night, and account for random effects across participants and sites.

For comparison to placebo, we report placebo-corrected change from baseline, which is the change from baseline of the treatment group minus the change from baseline of the placebo group. Through analysis of the EEG, we found that both at months 1 and 3 evaluations, patients treated with 50 and 25 mg of daridorexant had higher relative delta power during wake as compared to placebo (50 mg: month 1: **4.04%,***95% CI: 2.53%, 5.57%; p* < .001, month 3: **4.33%***95% CI*: 2.82%, 5.86%; *p* < .001; 25 mg: month 1: **2.56%,***95% CI: 1.05%, 4.08%; p* < .001, month 3: **3.03%,***95% CI: 1.53%, 4.53%; p < .001*; Figure 2). In addition, daridorexant reduced relative alpha power during wake compared to placebo at both follow-up evaluations (50 mg: month 1: **−1.73%***95% CI:−2.38%, −1.07%; p < .001,* month 3: **−2.08%**, *95% CI: −2.76%, −1.39%; p < .001;* 25 mg: month 1: **−1.51%,***95% CI: −2.18%, −0.84%; p* < .001, month 3: **−1.57%,***95% CI: −2.27%, −0.87%; p < .001*). No significant changes in relative delta or alpha power were observed for the daridorexant 25 and 50 mg dose groups when compared to baseline.

At months 1 and 3, respectively, 50 mg daridorexant reduced relative beta power compared to baseline during wake (**−1.16%,***95% CI:−2.03* %, −0.28%; *p* = .010 and **−1.08%***95% CI: −1.91%, −0.24%; p = .011*) and N1 **(−0.67%,***95% CI: −1.13%, −0.20%; p* = .005 and **−0.68%,***95% CI: −1.05%, −0.32%; p < .001*). When compared to placebo, relative beta power was also found to be significantly reduced in the 50 mg daridorexant group at both months 1 and 3 during wake (**−1.26%,***95% CI: −1.83%, −0.70; p < .001* and **−1.34%,***95% CI*:−1.92%, −0.75%, *p* < .001) and N1 **(−0.38%***95% CI: −0.72%, −0.03%, p = .034* and **−0.47%,***95% CI:−0.83, −0.11%, p = .010*). 25 mg of daridorexant did not significantly change relative beta power during Wake or N1 as compared to baseline and placebo, at either timepoint.

Of note, none of the analyzed spectral bands were statistically different from placebo or baseline in N2, N3, or REM sleep for both 50 and 25 mg groups.

**Figure 3.**
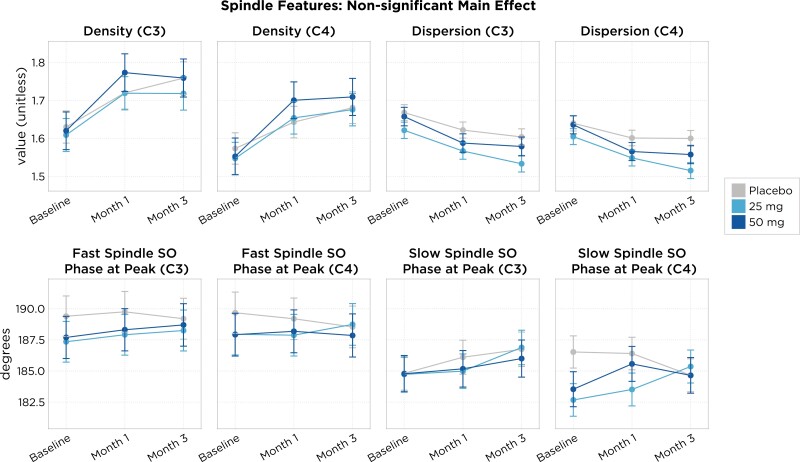
Shown are the mean (points) and 95% CI (shaded region) at each time point (*x*-axis) and treatment arm (colors). The main effect model showed a lack of statistical significance (*p* > .05), indicating that the treatment does not exert a measurable impact on the feature of interest when accounting for covariates such as age, sex, month, and night, as well as random effects for participants and sites.

Analysis of sleep spindles showed that treatment with daridorexant 25 and 50 mg did not significantly change either spindle density, dispersion, or phase-coupling (slow oscillation phase at spindle peak) characteristics, when compared to either baseline or the placebo group (Figure 3).

### WESI score changes across treatment groups

**Figure 4.**
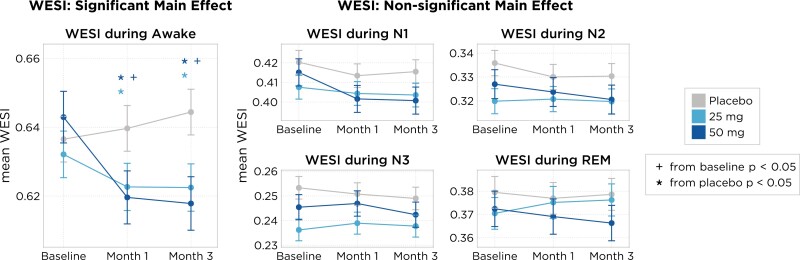
Shown are the mean WESI model predictions (*y*-axis) and 95% CIs (shaded region) at each evaluation (*x*-axis) and treatment arm (colors). The main effect model shows either a statistically significant effect (*p* < .05), indicating that treatment enhances the feature of interest, or a lack of statistical significance, suggesting that treatment does not have a measurable impact.

To determine whether changes related to daridorexant treatment could indicate less wake-like EEG features across the recording period, we employed a WESI measure using the aforementioned spectral bands. Relative to the placebo group, both daridorexant 50 and 25 mg decreased WESI scores during wake, at months 1 and 3 (Figure 4-left). However, relative to baseline, only daridorexant 50 mg significantly decreased WESI scores during Wake at both evaluations (−0.023 scores, *95%* CI: −0.042, −0.005, *p* = .014 at month 1 and −0.025 scores *95%* CI: −0.042, −0.008, *p* = .004 at month 3). Finally, WESI was slightly reduced across all sleep stages but did not differ significantly (*p* > .05) during N1, N2, N3, and REM sleep after daridorexant 50 or 25 mg when compared to baseline or placebo (Figure 4-center and right).

## Discussion

Accumulating evidence from previous studies has pointed to hyperarousal likely being the main factor contributing to the poor sleep quality and reduced sleep duration experienced by patients with chronic insomnia disorder [4, 5, 42, 43]. In the current study, we found that by blocking the wake and arousal activity of the orexin/hypocretin system [44], the DORA daridorexant reduced multiple hyperarousal EEG signatures present during wake and N1 sleep stages in patients with chronic insomnia disorder.

In our study, daridorexant reduced relative beta and alpha EEG power, both markers of a higher aroused brain state [45], and was found to be elevated in patients with insomnia [5, 46]. In addition, we observed that daridorexant reduced wake-to-wake transitions, while simultaneously increasing the transition probability from wake to either N1 or N2 sleep, further supporting its sleep-promoting effect [11, 29]. These results suggest that by dampening orexin/hypocretin signaling, daridorexant is able to reduce neurophysiological markers of hyperarousal and facilitate the transition back to sleep during the night.

Delta power during NREM sleep periods is believed to reflect homeostatic sleep pressure that increases as a function of time spent awake [47], which is believed to be imbalanced in patients with insomnia. Interestingly, we did not observe changes in delta power (0.5–4.0 Hz) in N2 and N3 sleep. Instead, we did observe a treatment-dependent effect, specifically the increase in relative delta power during wake, although evidence regarding the biological significance of delta during wake has been less robustly characterized. These findings may suggest that this delta power increase during wake indicates more drowsiness favoring transitions back to sleep. Importantly, these observations were not associated with excessive sedation in the morning [29].

This is further supported by the findings that, in patients treated with daridorexant a decrease in hyperarousal signatures is consistently observed when considering the reduction in both the relative alpha and beta power and the WESI scores during wake. These diminished wake-like EEG features during treatment may represent the neurophysiological improvement in sleep onset and sleep maintenance observed in these patients in a clinical setting, specifically the reductions in LPS and WASO and subsequent increases in TST [29].

In the current study, two doses of daridorexant (25 and 50 mg) and changes from baseline at two different timepoints (months 1 and 3), were considered. Despite no formal comparison, we found that the effects were greater across all parameters assessed with the 50 mg dose, and it was the only group to show a significant decrease for specific sleep architecture features, such as relative beta power and WESI score, when compared to both placebo and baseline. These findings are in line with previous results from this chronic insomnia disorder patient cohort where 50 mg was the only dose to show significant improvements both in sleep parameters and daytime functioning. The significant benefit on the two primary PSG parameters (WASO and LPS), was observed after 1 month and maintained until month 3 [29].

Previously, in a study involving a different DORA medication, suvorexant, an increase in TST was observed to be driven predominantly by increases in N2 sleep [48]. Here we found increases in N1-to-N2 transition probabilities, which may reflect a similar phenomenon. Interestingly, in the current study, we identified that spindle properties were not significantly affected, underscoring that intrinsic N2 sleep properties remain unchanged after treatment. This is in contrast with reported findings on GABA-A receptor agonists, which have been shown to increase sleep spindle activity [49]. In fact, as sleep spindles originate in thalamic circuits mediated by GABAergic neurons within the thalamic reticular nucleus, it is expected that medications acting on this receptor could alter sleep spindle morphology and temporal distribution [50]. However, rather than promoting inhibitory neurotransmission, daridorexant acts as an antagonist to orexins excitatory effects, unlike benzodiazepines [51], suggesting that treatment with a DORA preserves normal N2 sleep architecture. Furthermore, this is supported by the fact that spindle activity is not influenced by the orexinergic system, as confirmed in patients with narcolepsy where spindle activity is not altered [52].

Orexin 1 and 2 receptors, play a key role in promoting arousal, maintaining wake, and regulating REM sleep. Through antagonism of these two receptors, DORAs may induce sleep by reducing overactive wake signals in patients with chronic insomnia disorder, therefore impacting less the fundamental characteristics of the different stages of sleep [27, 29, 53]. Further evidence for the effect of DORAs in reducing excessive wakefulness was found in studies on both suvorexant [28] and daridorexant [11], which showed a reduction in the number and duration of longer wake bouts across the 8-hour night. This shortening of long wake bouts with DORAs may reflect their capacity to attenuate hyperarousal and thus reduce nighttime wakefulness via dual inhibition of orexin receptors. Previously, suvorexant was shown to modulate EEG power during NREM sleep by reducing fast frequency power and increasing lower frequency power, although these effects were only observed on the first night of treatment and not sustained after 1 month of treatment [26]. However, it is important to note that the effects of suvorexant or lemborexant (an additional DORA medication) on spectral features during REM sleep and wake, have not been evaluated.

Our analysis indicates that treatment with daridorexant reduces markers of hyperarousal associated with excessive nighttime wakefulness. This conclusion is drawn from an extensive and comprehensive dataset from 2 global phase 3 clinical trials. However, there are limitations that should be addressed. First, insomnia is a complex and heterogeneous disorder, with certain patients experiencing a decrease in daytime function without reductions in objective TST [54]. In these patients, daridorexant might have different effects on the qualitative and quantitative aspects of sleep, whereas our dataset included only patients that had objective changes to their sleep (e.g. TST < 420 minutes) as assessed by PSG. Second, our current analyses used single-night aggregate values, pooling individually scored epochs of each sleep–wake stage to control for interindividual differences attributed to our large sample size. However, this approach does not capture dynamic changes that occur throughout the night, and thus limits its specificity. Particularly, it fails to assess the timing of EEG feature changes during wake after daridorexant treatment, as such the current results do not allow for differentiation as to whether these EEG changes occur predominantly before sleep onset, in the middle of the night, or before lights-off. Lastly, this is an exploratory analysis and as such all results are considered hypothesis-generating only. Despite this limitation, we consistently observed significant differences between treatment groups across multiple measures.

Analysis of different EEG features as presented in this post hoc study demonstrates that markers associated with hyperarousal observed in chronic insomnia disorder can be reduced by daridorexant, even after 3 months of treatment. Furthermore, these effects were more pronounced at the higher dose of 50 mg and were most apparent during wake periods of the night. Taken together, these results suggest that daridorexant improves sleep by reducing high arousal spectral characteristics of wake and by increasing transitions from wake into sleep, thus reducing the time spent in long wake bouts in patients with chronic insomnia disorder.

## Supplementary material

Supplementary material is available at *SLEEP* online.

zsae098_suppl_Supplementary_Materials

## Data Availability

In addition to Idorsia’s existing clinical trial disclosure activities, the company is committed to implementing the Principles for Responsible Clinical Trial Data Sharing jointly issued by the European Federation of Pharmaceutical Industries and Associations (EFPIA) and the Pharmaceutical Research and Manufacturers of America (PhRMA). Requests for data sharing, of any level, can be directed to clinical-trials-disclosure@idorsia.com for medical and scientific evaluation.

## References

[1] National Institutes of Health. National Institutes of Health State of the Science Conference statement on manifestations and management of chronic insomnia in adults, June 13-15, 2005. Sleep. 2005;28(9):1049–1057. doi: 10.1093/sleep/28.9.1049 16268373

[2] Ishak WW , Bagot K, Thomas S, et al. Quality of life in patients suffering from insomnia. Innov Clin Neurosci. 2012;9(10):13–26. http://www.ncbi.nlm.nih.gov/pubmed/3508958 PMC3508958 23198273

[3] American Psychiatric Association. Diagnostic and Statistical Manual of Mental Disorders. Fifth. United States of America: American Psychiatric Association; 2013. doi: 10.1176/appi.books.9780890425596

[4] Vgontzas AN , Fernandez-Mendoza J, Liao D, Bixler EO. Insomnia with objective short sleep duration: the most biologically severe phenotype of the disorder. Sleep Med Rev. 2013;17(4):241–254. doi: 10.1016/j.smrv.2012.09.005 23419741 PMC3672328

[5] Dressle RJ , Riemann D. Hyperarousal in insomnia disorder: current evidence and potential mechanisms. J Sleep Res. 2023;32(6):e13928. doi: 10.1111/jsr.13928 37183177

[6] Hein M , Senterre C, Lanquart J-P, et al. Hyperarousal during sleep in untreated primary insomnia sufferers: a polysomnographic study. Psychiatry Res. 2017;253:71–78. doi: 10.1016/j.psychres.2017.03.045 28364590

[7] Oh DY , Park SM, Choi SW. Daytime neurophysiological hyperarousal in chronic insomnia: a Study of qEEG. J Clin Med. 2020;9(11):3425. doi: 10.3390/jcm9113425 33114486 PMC7694040

[8] Perlis ML , Smith MT, Andrews PJ, Orff H, Giles DE. Beta/Gamma EEG activity in patients with primary and secondary insomnia and good sleeper controls. Sleep. 2001;24(1):110–117. doi: 10.1093/sleep/24.1.110 11204046

[9] Spiegelhalder K , Regen W, Feige B, et al. Increased EEG sigma and beta power during NREM sleep in primary insomnia. Biol Psychol. 2012;91(3):329–333. doi: 10.1016/j.biopsycho.2012.08.009 22960269

[10] Svetnik V , Snyder ES, Ma J, Tao P, Lines C, Herring WJ. EEG spectral analysis of NREM sleep in a large sample of patients with insomnia and good sleepers: effects of age, sex and part of the night. J Sleep Res. 2017;26(1):92–104. doi: 10.1111/jsr.12448 27634437

[11] Di Marco T , Scammell TE, Meinel M, et al. Number, duration, and distribution of wake bouts in patients with insomnia disorder: Effect of daridorexant and zolpidem. CNS Drugs. 2023;37(7):639–653. doi: 10.1007/s40263-023-01020-9 37477771 PMC10374812

[12] Nofzinger EA , Boysse DJ, Germain A, Price JC, Miewald JM, Kopfer DJ. Functional neuroimaging evidence for hyperarousal in insomnia. Am J Psychiatry. 2004;161(11):2126–2129. doi: 10.1176/appi.ajp.161.11.2126 15514418

[13] Sateia M , Buysse D, Krystal AD, Neubauer DN, Heald JL. Clinical practice guideline for the pharmacologic treatment of chronic insomnia in adults. J Clin Sleep Med. 2017;13(5):307–349. 27998379 10.5664/jcsm.6470 PMC5263087

[14] Edinger JD , Arnedt JT, Bertisch SM, et al. Behavioral and psychological treatments for chronic insomnia disorder in adults: an American Academy of Sleep Medicine clinical practice guideline. J Clin Sleep Med. 2021;17(2):255–262. doi: 10.5664/jcsm.8986 33164742 PMC7853203

[15] Riemann D , Espie CA, Altena E, et al. The European Insomnia Guideline: an update on the diagnosis and treatment of insomnia 2023. J Sleep Res. 2023;32(6):e14035. doi: 10.1111/jsr.14035 38016484

[16] Cervena K , Dauvilliers Y, Espa F, et al. Effect of cognitive behavioural therapy for insomnia on sleep architecture and sleep EEG power spectra in psychophysiological insomnia. J Sleep Res. 2004;13(4):385–393. doi: 10.1111/j.1365-2869.2004.00431.x 15560773

[17] Thomas A , Grandner M, Nowakowski S, Nesom G, Corbitt C, Perlis ML. Where are the behavioral sleep medicine providers and where are they needed? A geographic assessment. Behav Sleep Med. 2016;14(6):687–698. doi: 10.1080/15402002.2016.1173551 27159249 PMC5070478

[18] Ferini‐Strambi L , Auer R, Bjorvatn B, et al.; the European Sleep Foundation. Insomnia disorder: clinical and research challenges for the 21st century. Eur J Neurol. 2021;28(7):2156–2167. doi: 10.1111/ene.14784 33619858

[19] Soyka M , Wild I, Caulet B, Leontiou C, Lugoboni F, Hajak G. Long-term use of benzodiazepines in chronic insomnia: a European perspective. Front Psychiatry. 2023;14:1212028. doi: 10.3389/fpsyt.2023.1212028 37599882 PMC10433200

[20] Monti JM , Spence DW, Pandi-Perumal SR, Langer SZ, Hardeland R. Pharmacotherapy of Insomnia: focus on zolpidem extended release. Clin Med. Therapeut. 2009;1:CMT.S2040. doi: 10.4137/cmt.s2040

[21] Patat A , Trocherie S, Thebault JJ, et al. EEG profile of intravenous zolpidem in healthy volunteers. Psychopharmacology (Berl). 1994;114(1):138–146. doi: 10.1007/BF02245455 7846196

[22] Declerck AC , Ruwe F, O’Hanlon JF, Wauquier A. Effects of zolpidem and flunitrazepam on nocturnal sleep of women subjectively complaining of insomnia. Psychopharmacology (Berl). 1992;106(4):497–501. doi: 10.1007/bf02244821 1579623

[23] de Lecea L , Kilduff TS, Peyron C, et al. The hypocretins: hypothalamus-specific peptides with neuroexcitatory activity. Proc Natl Acad Sci USA. 1998;95(1):322–327. doi: 10.1073/pnas.95.1.322 9419374 PMC18213

[24] Sakurai T , Amemiya A, Ishii M, et al. Orexins and orexin receptors: a family of hypothalamic neuropeptides and G protein-coupled receptors that regulate feeding behavior. Cell. 1998;92(4):573–585. doi: 10.1016/s0092-8674(00)80949-6 9491897

[25] Sakurai T. The neural circuit of orexin (hypocretin): maintaining sleep and wakefulness. Nat Rev Neurosci. 2007;8(3):171–181. doi: 10.1038/nrn2092 17299454

[26] Snyder E , Ma J, Svetnik V, et al. Effects of suvorexant on sleep architecture and power spectral profile in patients with insomnia: analysis of pooled phase 3 data. Sleep Med. 2016;19:93–100. doi: 10.1016/j.sleep.2015.10.007 27198953

[27] Moline M , Zammit G, Cheng J, Perdomo C, Kumar D, Mayleben D. Comparison of the effect of lemborexant with placebo and zolpidem tartrate extended release on sleep architecture in older adults with insomnia disorder. J Clin Sleep Med. 2021;17:1167–1174. doi:10.5664/jcsm.9150 33590823 PMC8314653

[28] Svetnik V , Snyder ES, Tao P, et al. Insight into reduction of wakefulness by suvorexant in patients with insomnia: analysis of wake bouts. Sleep. 2018;41(1). doi: 10.1093/sleep/zsx178 29112763

[29] Mignot E , Mayleben D, Fietze I, et al.; investigators. Safety and efficacy of daridorexant in patients with insomnia disorder: results from two multicentre, randomised, double-blind, placebo-controlled, phase 3 trials. Lancet Neurol. 2022;21(2):125–139. doi: 10.1016/S1474-4422(21)00436-1 35065036

[30] Dauvilliers Y , Zammit G, Fietze I, et al. Daridorexant, a new dual orexin receptor antagonist to treat insomnia disorder. Ann Neurol. 2020;87(3):347–356. doi: 10.1002/ana.25680 31953863

[31] Zammit G , Dauvilliers Y, Pain S, Sebök Kinter D, Mansour Y, Kunz D. Daridorexant, a new dual orexin receptor antagonist, in elderly subjects with insomnia disorder. Neurology. 2020;94(21):e2222–e2232. doi: 10.1212/WNL.0000000000009475 32341187

[32] Morin CM , Belleville G, Bélanger L, Ivers H. The Insomnia severity index: psychometric indicators to detect insomnia cases and evaluate treatment response. Sleep. 2011;34(5):601–608. doi: 10.1093/sleep/34.5.601 21532953 PMC3079939

[33] Sheehan DV , Lecrubier Y, Sheehan KH, et al. The Mini-International Neuropsychiatric Interview (M.I.N.I.): the development and validation of a structured diagnostic psychiatric interview for DSM-IV and ICD-10. J Clin Psychiatry. 1998;59(suppl 2) :22–33;quiz 34. http://www.ncbi.nlm.nih.gov/pubmed/9881538 9881538

[34] Berry RB , Brooks R, Gamaldo CE, Harding SM, Lloyd RM, Marcus CL, et al. The AASM manual for the scoring of sleep and associated events: rules, terminology and technical specifications, version 2.2. Am Acad Sleep Med. 2015. www.aasmnet.org

[35] Wei Y , Colombo MA, Ramautar JR, et al. Sleep stage transition dynamics reveal specific stage 2 vulnerability in insomnia. Sleep. 2017;40(9). doi: 10.1093/sleep/zsx117 28934523

[36] Thomson DJ. Spectrum estimation and harmonic analysis. Proc IEEE. 1982;70(9):1055–1096. doi: 10.1109/proc.1982.12433

[37] Chikhi S , Matton N, Blanchet S. <scp>EEG</scp> power spectral measures of cognitive workload: a meta‐analysis. Psychophysiology. 2022;59(6):e14009. doi: 10.1111/psyp.14009 35128686

[38] Ernst Niedermeyer, M.D; Fernando Lopes Da Silva, M.D. P. Electroenecephalography, Basic Principles, Clinical Applications, and Related Fields. Fifth Edit. Philadelphia, PA: Lippincott WIlliams & Wilkins; 2005.

[39] Purcell SM , Manoach DS, Demanuele C, et al. Characterizing sleep spindles in 11,630 individuals from the National Sleep Research Resource. Nat Commun. 2017;8(1):15930. doi: 10.1038/ncomms15930 28649997 PMC5490197

[40] Warby SC , Wendt SL, Welinder P, et al. Sleep-spindle detection: crowdsourcing and evaluating performance of experts, non-experts and automated methods. Nat Methods. 2014;11(4):385–392. doi: 10.1038/nmeth.2855 24562424 PMC3972193

[41] Younes M , Ostrowski M, Soiferman M, et al. Odds ratio product of sleep EEG as a continuous measure of sleep state. Sleep. 2015;38(4):641–654. doi: 10.5665/sleep.4588 25348125 PMC4355904

[42] Palagini L , Geoffroy PA, Balestrieri M, et al. Current models of insomnia disorder: a theoretical review on the potential role of the orexinergic pathway with implications for insomnia treatment. J Sleep Res. 2023;32(4):e13825. doi:10.1111/jsr.13825 36786121

[43] Kay D , Buysse D. Hyperarousal and beyond: New Insights to the pathophysiology of insomnia disorder through functional neuroimaging studies. Brain Sci. 2017;7(12):23. doi: 10.3390/brainsci7030023 28241468 PMC5366822

[44] De Luca R , Nardone S, Grace KP, et al. Orexin neurons inhibit sleep to promote arousal. Nat Commun. 2022;13(1):4163. doi: 10.1038/s41467-022-31591-y 35851580 PMC9293990

[45] Palva S , Palva JM. New vistas for α-frequency band oscillations. Trends Neurosci. 2007;30(4):150–158. doi: 10.1016/j.tins.2007.02.001 17307258

[46] Zhao W , Van Someren EJW, Li C, et al. EEG spectral analysis in insomnia disorder: a systematic review and meta-analysis. Sleep Med Rev. 2021;59(2):101457. doi: 10.1016/j.smrv.2021.101457 33607464

[47] Hubbard J , Gent TC, Hoekstra MMB, et al. Rapid fast-delta decay following prolonged wakefulness marks a phase of wake-inertia in NREM sleep. Nat Commun. 2020;11(1):3130. doi: 10.1038/s41467-020-16915-0 32561733 PMC7305232

[48] Herring WJ , Snyder E, Budd K, et al. Orexin receptor antagonism for treatment of insomnia: a randomized clinical trial of suvorexant. Neurology. 2012;79(23):2265–2274. doi: 10.1212/WNL.0b013e31827688ee 23197752

[49] Leong CWY , Leow JWS, Grunstein RR, et al. A systematic scoping review of the effects of central nervous system active drugs on sleep spindles and sleep-dependent memory consolidation. Sleep Med Rev. 2022;62:101605. doi: 10.1016/j.smrv.2022.101605 35313262

[50] Fernandez LMJ , Lüthi A. Sleep spindles: mechanisms and functions. Physiol Rev. 2020;100(2):805–868. doi: 10.1152/physrev.00042.2018 31804897

[51] de Mendonça FMR , de Mendonça GPRR, Souza LC, et al. Benzodiazepines and sleep architecture: a systematic review. CNS Neurol Disord Drug Targets. 2023;22(2):172–179. doi: 10.2174/1871527320666210618103344 34145997

[52] Christensen JAE , Nikolic M, Hvidtfelt M, Kornum BR, Jennum P. Sleep spindle density in narcolepsy. Sleep Med. 2017;34:40–49. doi: 10.1016/j.sleep.2017.02.022 28522097

[53] O-ZJ Kron J , Keenan RJ, Hoyer D, Jacobson LH. Orexin receptor antagonism: normalizing sleep architecture in old age and disease. Annu Rev Pharmacol Toxicol. 2024;64(1):359–386. doi:10.1146/annurev-pharmtox-040323-031929 37708433

[54] Benbir Şenel G , Aydın O, Tanrıöver Aydın E, Bayar MR, Karadeniz D. Changes in sleep structure and sleep spindles are associated with the neuropsychiatric profile in paradoxical insomnia. Int J Psychophysiol. 2021;168:27–32. doi: 10.1016/j.ijpsycho.2021.07.626 34331959

