# Supplementary material for: Effect of daridorexant on sleep architecture in patients with chronic insomnia disorder: a pooled post hoc analysis of two randomized phase 3 clinical studies
Source: Sleep. 2024 Apr 22;47(11):zsae098. doi: 10.1093/sleep/zsae098 (PMC11543623; doi:10.1093/sleep/zsae098)
Supplement: zsae098_suppl_Supplementary_Materials [file zsae098_suppl_supplementary_materials.docx]

**Effect of daridorexant on sleep architecture in patients with chronic insomnia disorder – An analysis of two pooled Phase 3 studies**

Tobias Di Marco^1,2^, Ina Djonlagic^3^, Yves Dauvilliers^4^, Kolia Sadeghi^5^, David Little^5^, Alexandre N. Datta^6^, Jeffrey Hubbard^1^, Göran Hajak^7^, Andrew Krystal^8^, Antonio Olivieri^1^, Liborio Parrino^9^, Corey B. Puryear^5^, Gary Zammit^10^, Jacob Donoghue^5*^, Thomas E. Scammell^3*^

1. Idorsia Pharmaceuticals Ltd, Allschwil, Switzerland

2. Department of Clinical Research, University of Basel, Schanzenstrasse 55, 4031 Basel,

3. Department of Neurology, Beth Israel Deaconess Medical Center, Boston, MA, United States

4. Centre National de Référence Narcolepsie, Unité du Sommeil, CHU Montpellier, Hôpital Gui–de–Chauliac, Université de Montpellier, INSERM INM, Montpellier, France

5. Beacon Biosignals, Inc., Boston, MA, United States

6. University Children’s Hospital Basel, Basel, Switzerland

7. Social Foundation Bamberg, Department of Psychiatry, Psychosomatic Medicine and Psychotherapy, Bamberg, Germany

8. University of California, San Francisco, CA, USA

9. University of Parma, Department of Medicine and Surgery, Parma, Italy

10. Clinilabs Drug Development Corporation, New York, USA

*Dual co–senior authors, equal contributions

**Corresponding authors:**

1. Tobias Di Marco

Hegenheimermattweg 91, 4123 Allschwil, Switzerland,

E–mail: [tobias.dimarco@unibas.ch](mailto:tobias.dimarco@unibas.ch)

2. Thomas E. Scammel
Beth Israel Deaconess Medical Center, Boston, MA, United States 
E-mail: tscammel@bidmc.harvard.edu

**Target journal:** Sleep

# **Supplement**:


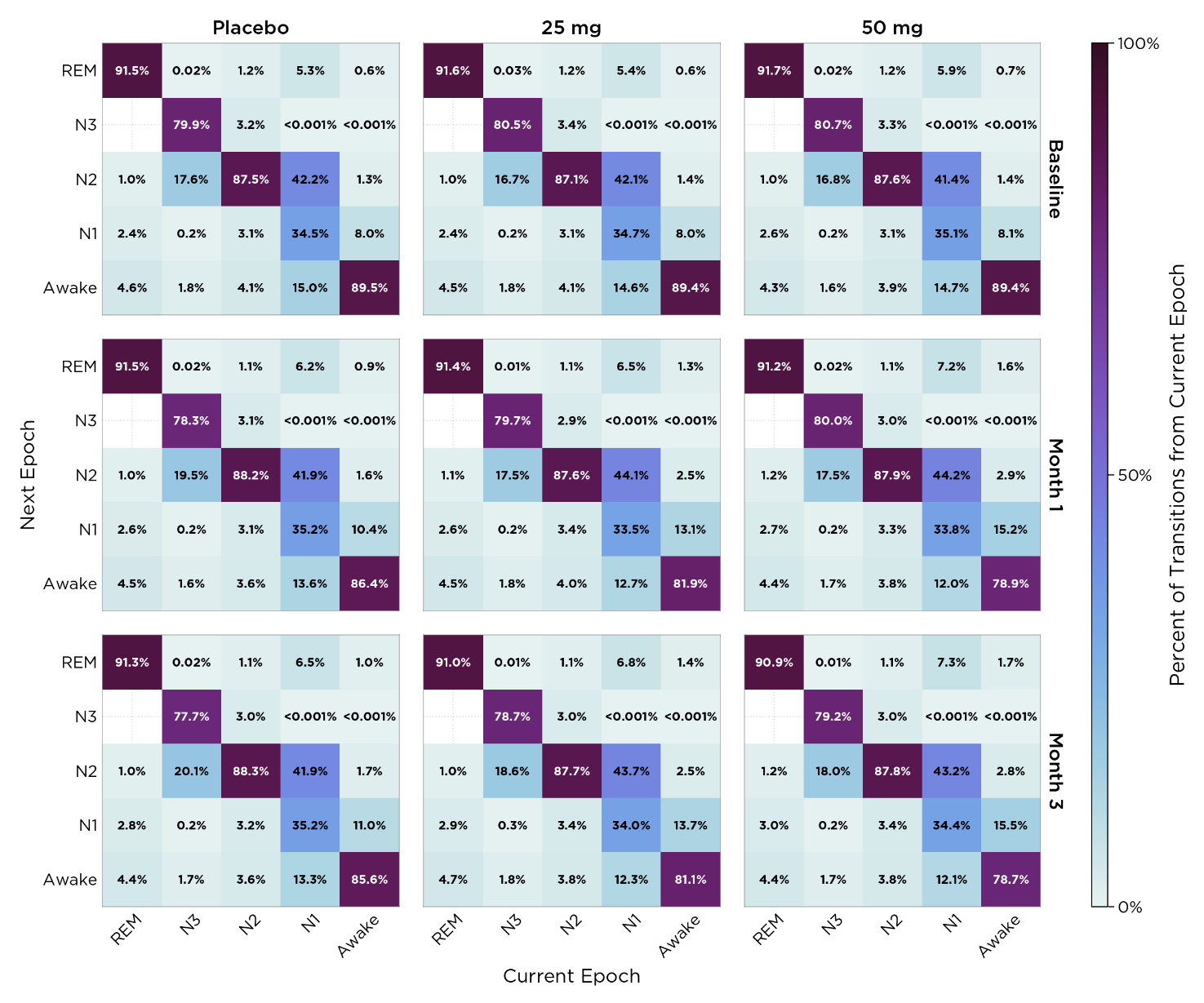


Supplement 1 Sleep–Wake transitions: Matrices for all possible sleep stage transitions. Each box shows the model–estimated transition probability (between 0 and 1) for Baseline, Month 1, and 3 evaluations, respectively. Blank box indicates an absence of that sleep stage transition in either group.
